# The NOD (Naltrexone for Overdose Prevention) study protocol: a pilot randomized controlled trial of intramuscular naltrexone for opioid overdose prevention among people who use stimulants living with or at risk for HIV

**DOI:** 10.1186/s13722-025-00623-5

**Published:** 2025-12-15

**Authors:** Ayesha Appa, Xochitl Luna Marti, Stefan Baral, Steven Shoptaw, Alexander R. Bazazi, Matthew A. Spinelli, Dave Glidden, Monica Gandhi, Phillip Coffin

**Affiliations:** 1 University of California, San Francisco, San Francisco, USA; 2 San Francisco Department of Public Health, San Francisco, USA; 3 Johns Hopkins University, Baltimore, USA; 4 University of California, Los Angeles, Los Angeles, USA

**Keywords:** Substance use disorder, Drug users, Central nervous system stimulants, Overdose

## Abstract

**Background:**

Drug-related mortality in the United States continues to transform, marked by a surge in overdose deaths involving both fentanyl and stimulants. This pattern presents specific risks for people at risk for and living with HIV, who have high rates of stimulant use and disproportionate risk of opioid overdose due to unintentional fentanyl use.

**Study objectives:**

This pilot randomized controlled trial evaluates intramuscular naltrexone as a novel biomedical strategy for opioid overdose prevention among people who use stimulants living with or at risk for HIV. The primary outcome is acceptability; secondary outcomes include effectiveness and safety; and an exploratory outcome characterizes patterns of opioid exposure among people reporting only stimulant use.

**Intervention design:**

: The NOD (Naltrexone for Overdose Prevention) Study will randomize 100 participants 1:1 to receive intramuscular naltrexone (380 mg) every 4 weeks or usual care for 24 weeks. Both arms will receive harm reduction services for overdose prevention including intranasal naloxone and safer consumption supplies.

**Methods:**

Acceptability (primary outcome) will be assessed quantitatively through retention at 24 weeks and proportion of on-time injections, and qualitatively through in-depth interviews (*n* = 35). Effectiveness (secondary outcome) includes incident opioid overdose events requiring medical attention or community naloxone. Safety includes grade 2 or higher adverse events. Opioid exposure patterns (exploratory outcome) will be assessed through monthly urine toxicology, hair samples analyzed via liquid chromatography/mass spectrometry, and Timeline Followback interviews. Analysis will use Kaplan-Meier methods for time-to-event outcomes and mixed methods for implementation evaluation.

**Discussion:**

This study leverages the concept of HIV pre-exposure prophylaxis to the prevention of overdose by evaluating the viability of naltrexone as protection against opioid overdose. Success would demonstrate acceptability of long-acting opioid overdose prevention in people who use stimulants, while providing critical insights into implementation and impact of naltrexone as overdose prevention, as well as evaluating patterns of unintentional opioid use.

## Introduction

The epidemiology of drug-related mortality in the United States has undergone a dramatic transformation, marked by a rise in overdose deaths involving both fentanyl and stimulants. From 2010 to 2021, the proportion of overdose deaths involving both fentanyl and stimulants like methamphetamine increased 60-fold [1]. In San Francisco, over 40% of medically-attended nonfatal opioid overdose events occurred among people who reported no intentional opioid use, with most intending to use only stimulants [2]. Unintentional opioid use may result from fentanyl contamination of stimulants, accidental use of fentanyl given its similar appearance to stimulants, or use of cross-contaminated smoking equipment. This pattern of unintentional fentanyl use presents a critical challenge to existing overdose prevention strategies.

People living with HIV (PWH) and those at risk for HIV infection face heightened vulnerability in this evolving crisis. Stimulant use is highly prevalent, with methamphetamine and cocaine use rates ranging from 15 to 30% in general HIV cohorts and exceeding 80% in a cohort of PWH experiencing homelessness [3–5]. Although PWH have had historically lower rates of opioid use disorder (as compared to other substance use disorders), life expectancy among PWH in some regions has decreased related to the overdose crisis, suggesting unintentional fentanyl use could be playing a role [4, 6]. The analysis of substances in hair is emerging as a powerful biomarker to either detect or quantify patterns of substance use over time [7, 8]. Recent biomarker studies by our group using hair analyses demonstrated that 22% of gay men and other men who have sex with men at risk for HIV or living with HIV had hair samples positive for fentanyl, despite denying opioid use [9]. 

Current overdose prevention strategies demonstrate significant limitations in addressing unintentional fentanyl use. While community drug checking services and fentanyl test strips offer some protection, legal and implementation barriers and technical limitations restrict their protective potential [10, 11]. Traditional interventions such as opioid agonist treatments effectively prevent overdose in people with opioid use disorder, but are not appropriate for individuals exclusively using stimulants. The increasing number of overdose deaths related to both fentanyl and stimulants creates an urgent need for novel prevention approaches.

**Fig. 1:**
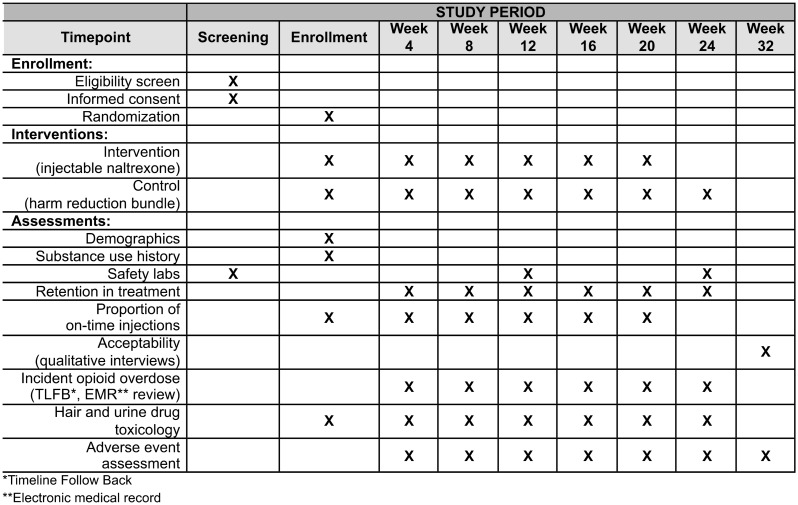
NOD study measures & procedures

Naltrexone, an opioid receptor antagonist with an established safety profile in treating alcohol and opioid use disorders, represents a potential but unexplored tool for overdose prevention in this context [12]. Naltrexone functions as a competitive antagonist at µ-opioid receptors, the primary site of action for fentanyl’s respiratory depressant effects. By occupying these receptors with high affinity and long duration (approximately 4 weeks for the long-acting formulation), naltrexone prevents fentanyl from binding and exerting its potentially fatal respiratory depressant effects [13]. This mechanism may be particularly relevant for opioid-naïve individuals who lack tolerance to opioid-induced respiratory depression, in which small amounts of inadvertent fentanyl could result in fatal overdose. While the role of naltrexone in addiction treatment is well-documented, its potential as a preventive intervention for people who use stimulants at risk of unintentional opioid exposure remains unstudied. Monthly intramuscular naltrexone could offer a novel biomedical approach to overdose prevention, particularly valuable for PWH and others who use stimulants but do not intentionally use opioids and lack opioid tolerance. This approach aligns with lessons learned from HIV prevention, where long-acting interventions have proven more effective than those requiring daily adherence or point-of-use decision-making [14]. However, risks and benefits of this approach are unknown (Fig. 1).

### NOD study objectives

The Naltrexone for Overdose Prevention (NOD) study is a pilot randomized controlled trial designed to evaluate intramuscular naltrexone as a novel biomedical strategy for opioid overdose prevention among people who use stimulants living with or at risk for HIV, with the following outcomes:

1. The primary outcome is acceptability of intramuscular naltrexone as opioid overdose prevention, assessed quantitatively by retention at 24 weeks and proportion of on-time injections, and qualitatively through sequential explanatory in-depth interviews.
2. Secondary outcomes include: (A) effectiveness, evaluated by incident opioid overdose events and (B) safety.
3. An exploratory objective is to characterize patterns of opioid exposure using both short-term (urine) and long-term (hair) biomarkers among people who report stimulant use without intentional opioid use, providing insight into the scope and frequency of unintentional fentanyl exposure in this population.

## Methods

### NOD study design

This 24-week pilot randomized controlled trial will evaluate intramuscular naltrexone for opioid overdose prevention among individuals living with or at risk for HIV who use stimulants. The study will enroll 100 participants randomized 1:1 to receive either monthly intramuscular naltrexone (380 mg) or usual care, and both groups will receive harm reduction services.

#### Study setting and population

The study will be conducted at the San Francisco Department of Public Health, with targeted recruitment from HIV prevention and treatment settings. Eligible participants must: (1) be living with HIV or at risk for HIV through condomless serodiscordant or unknown sex or shared injection equipment, (2) use cocaine or methamphetamine at least 10 days per month AND either meet criteria for stimulant use disorder *or* have positive urine toxicology testing for methamphetamine or cocaine in past 6 months, (3) be English-speaking, and (4) be 18 years or older. We will *exclude* individuals with any prescribed or non-prescribed opioid use, use of extended-release naltrexone within the prior 30 days, planned surgery in the next six months, moderate to severe liver disease, pregnancy or breastfeeding, bleeding risk, or naltrexone hypersensitivity.

#### Intervention

Participants randomized to the intervention arm will receive monthly intramuscular naltrexone 380 mg administered in the ventrogluteal region. Both intervention and usual care arms will receive standard harm reduction services including naloxone, safer consumption supplies, and fentanyl test strips. All participants will undergo monthly clinical assessments and biomarker collection for the evaluation of substance use throughout the 24-week study period.

#### Randomization and blinding

Following eligibility confirmation and obtaining written informed consent, participants will be randomized 1:1 to either intramuscular naltrexone or usual care using a computer-generated randomization sequence with random block sizes. Participants and clinicians will not be blinded to treatment assignment, but laboratory personnel conducting biomarker analyses will remain blinded to treatment allocation.

#### Baseline assessments

At screening, participants will undergo comprehensive evaluation including medical history, physical examination, and DSM-V assessment for substance use disorders [15]. Laboratory testing will include complete blood count, comprehensive metabolic panel, and HIV testing for those reporting HIV-negative status. For participants who report living with HIV, status will be confirmed through medical records or documentation. Prior to enrollment, participants will complete informed consent that includes detailed discussion of randomization, medical risks and medical record review, and education regarding the investigational nature of naltrexone for overdose prevention.

#### Intervention arm & standard care components

Participants randomized to the intervention arm will receive monthly intramuscular naltrexone (380 mg) administered in the ventrogluteal region. Prior to the initial injection, participants will undergo point-of-care urine testing to confirm absence of opioids and prevent precipitated withdrawal. For participants with positive fentanyl testing who deny intentional use, or in cases of clinical uncertainty regarding unintentional exposure, we offer an intranasal naloxone challenge before long-acting naltrexone administration. The choice of ventrogluteal injection site is based on previous research suggesting better tolerability at this site compared to dorsogluteal administration [16]. Both intervention and usual care arms will receive harm reduction supplies. This includes intranasal naloxone (4 mg, two doses), participant-selected safer consumption supplies tailored to route of administration, and fentanyl test strips with instructions to minimize false positives.

#### Study visits and clinical monitoring

Following enrollment, participants attend study visits every 4 weeks through week 24, compensated $50 per visit. Each visit includes a symptom-driven physical examination, vital signs monitoring, and adverse event assessment. Laboratory monitoring includes complete blood count and comprehensive metabolic panel at weeks 12 and 24.

#### Post-study transition

At study conclusion, all participants, including those who discontinue early, will be offered assistance in receiving provider-prescribed naltrexone through their primary care providers. For participants receiving HIV care at San Francisco General Hospital’s Ward 86 clinic who are on long-acting injectable antiretroviral therapy, we offer coordination of naltrexone administration with their routine HIV care visits. This ensures continuity of care beyond the study period for participants who find the intervention beneficial.

#### Protocol modifications and discontinuation

The protocol includes specific criteria for individual participant discontinuation and study-wide stopping rules. Individual discontinuation may occur due to participant request, unacceptable toxicity, or need for full-agonist opioids. Study-wide stopping rules include observation of unanticipated adverse effects or emergence of new safety information. In addition to the UCSF Institutional Review Board, the UCLA Data Safety Monitoring Board reviews all serious adverse events and provides recommendations annually regarding study continuation.

### Assessment & data collection

#### Overview

The study will assess outcomes across three categories, aligned with our primary, seconday, and exploratory objectives. The primary outcome is acceptability of intramuscular naltrexone, evaluated through both quantitative implementation metrics and qualitative interviews with participants in both arms. Secondary outcomes include effectiveness (incident opioid overdose events requiring medical attention or community naloxone administration) and and safety including grade 2 or higher adverse events. The exploratory outcome characterizes patterns of opioid exposure via monthly point-of-care urine toxicology, hair analysis for substance exposure via liquid chromatography-tandem mass spectrometry (LC-MS/MS)-based methods, and Timeline Followback interviews [17]. 

#### Primary outcome: acceptability

The primary outcome is acceptability of intramuscular naltrexone as opioid overdose prevention, assessed through both quantitative and qualitative measures. Quantitatively, we will evaluate the following implementation outcomes: (1) the proportion of participants recruited versus enrolled, (2) time to goal enrollment, (3) number of visits completed per participant, (4) retention in treatment at 24 weeks, and (5) the proportion of on-time injections during the study period. We will consider 50% of enrolled participants completing 3 or more injections the minimum standard for adequate intervention uptake.

For qualitative evaluation, we will conduct sequential explanatory in-depth interviews with up to 35 individuals purposively sampled across levels of engagement, including participants from both study arms and eligible individuals who declined participation. This sample size was strategically chosen to ensure comprehensive thematic saturation while capturing a broad spectrum of participant experiences with long-acting injectable naltrexone as an overdose prevention strategy. The interviews will explore multiple dimensions, including general acceptability factors like intervention burden and perceived effectiveness; intramuscular naltrexone-specific factors, addressing subjective experiences of ongoing substance use, risk compensation, and trust in long-acting injectable medications; and HIV-specific factors, including experiences with multiple injectable medications for HIV and SUDs. Additional topics include perceived frequency of unintentional opioid exposure among people using stimulants, proposed mechanisms of exposure, and views on harm reduction strategies.

#### Secondary outcomes: effectiveness and safety outcomes

The secondary outcome of effectiveness is incident opioid overdose, defined as participant-reported overdose responding to community-administered naloxone, overdose requiring emergency medical services, or fatal overdose. Fatal overdoses will be ascertained through: (1) review of the San Francisco Medical Examiner records, (2) review of emergency medical services records and hospital systems within San Francisco, and (3) participant emergency contacts who will be contacted if participants miss consecutive study visits. Secondary safety outcomes include the proportion of participants experiencing grade 2 or higher adverse events at least possibly related to study medication, including injection site reactions, hepatic abnormalities, and other adverse events. Grade 2 adverse events are defined as moderate events requiring minimal intervention (e.g., nausea), while grades 3–5 represent severe, life-threatening, or fatal events requiring significant intervention (e.g., opioid overdose) [18]. 

#### Exploratory outcome: exposure assessment and biomarkers

To characterize patterns of opioid exposure, we will collect both short-term and long-term biomarkers at monthly visits. Urine will be tested using a CLIA-waived enzyme immunoassay. Hair samples will be analyzed at the UCSF Hair Analytical Laboratory using a validated panel that enables assessment of substance exposure (e.g. all opioids, cocaine, methamphetamine) via LC-MS/MS-based methods over sequential monthly intervals. These objective measures will be paired with Timeline Followback interviews to assess discordance between self-reported and biological measures of exposure.

#### Follow-up assessments

Monthly follow-up visits will include clinical assessment, adverse event monitoring, and collection of biomarkers. At weeks 12 and 24, participants will undergo additional laboratory testing including complete blood count and comprehensive metabolic panels. All participants, regardless of study completion or early discontinuation, will be offered assistance to receive provider-prescribed naltrexone at study conclusion which is possible via public insurance in California. A final assessment at week 32 will evaluate incident overdoses and adverse events via telephone interview.

### Analysis plans

#### Sample size and power

The sample size calculation is based on the primary quantitative outcome of retention (acceptability). With 50 participants per arm, the study has 85% power to detect a 25% difference in retention between groups (α = 0.05). The baseline incidence of unintentional opioid overdose is unknown, precluding formal power calculations for effectiveness outcomes. However, prior research in our population of intereset suggests opioid overdose mortality rates of approximately 5% over 12 months among people living with HIV who use stimulants, suggesting our sample should provide adequate sensitivity to detect preliminary effectiveness signals [19]. Observation of 4.72 incident overdoses in the usual care group (12% over 24 weeks) and 0 overdoses in the intervention group would provide 80% power to reject the null hypothesis of equivalent overdose risk between groups.

#### Implementation analysis (Primary)

We will use the RE-AIM framework to guide analysis of implementation outcomes, focusing on Reach, Effectiveness, and Implementation. Reach will be evaluated through the proportion of screened to enrolled participants. Effectiveness analyses are detailed below. Implementation will be assessed through the proportion of on-time injections, defining adequate uptake as ≥ 3 injections in ≥ 50% of participants, and Kaplan-Meier estimates of time to discontinuation. Cox proportional hazards models will examine attrition risk by pre-specified subgroups including demographics, housing status, and concurrent medications.

#### Effectiveness analysis (Secondary)

Time-to-event analysis using Kaplan-Meier methods will evaluate cumulative probability of first opioid overdose. Cox proportional hazards models will estimate overdose risk adjusting for key covariates including age, race/ethnicity, housing status, and HIV status. Safety analyses will compare the proportion of adverse events between arms using exact methods for binary outcomes and regression models for continuous safety measures.

#### Exposure pattern analysis (Exploratory)

To characterize unintentional opioid exposure, we will implement a multi-level analytical approach comparing self-reported use with biological markers, accounting for their different detection windows. First, we will calculate the prevalence of opioid/fentanyl-positive specimens among participants consistently not reporting intentional opioid use on Timeline Followback (TLFB) interviews.

For urine analyses (with a typical 2–7 day detection window), we will align positive results with the preceding 7-day period in TLFB data to determine potential discordance. This short detection window provides information about recent exposure but may miss intermittent use. We will calculate point prevalence of discordance at each monthly visit, as well as cumulative incidence over the study period. For hair analyses (capturing approximately 30-day exposure history per 1 cm segment) [20], we will implement segmental analysis corresponding to the study’s monthly visit structure. For each monthly segment, we will compare hair results with the corresponding month of TLFB data to identify discordance over longer timeframes. We will calculate both the prevalence of any detectable exposure and quantify concentration levels.

#### Missing data and sensitivity analyses

Primary analyses will use intention-to-treat principles. Multiple imputation will address missing data when appropriate. Sensitivity analyses will include per-protocol analysis and evaluation of different operational definitions of retention and exposure. We will conduct interim analyses for safety monitoring but not for effectiveness unless advised by the appointed Data Safety and Monitoring Board.

## Discussion

The profound loss of life from opioid overdose is particularly concerning when it occurs among individuals who did not intentionally use opioids. Implementing novel overdose prevention strategies for people who use stimulants will be a critical step towards addressing the evolving overdose crisis. We describe an innovative approach that adapts the concept of pre-exposure prophylaxis from HIV prevention to overdose prevention, potentially offering protection against unintentional opioid use. Implementation in a public health research setting will provide foundational data to inform future integration into clinical care, while inclusion of both PWH and those at risk provides an opportunity to reach a population with high rates of stimulant use and demonstrated vulnerability to intentional and unintentional fentanyl use.

The study design offers several methodological strengths. The use of both short-term and long-term biomarkers provides unprecedented insight into patterns of unintentional opioid exposure among people who use stimulants. This comprehensive biological monitoring, combined with detailed qualitative assessment, will help characterize the scope of unintentional use while centering community perspectives on acceptability and implementation. As a pilot trial, this study will provide critical data on baseline overdose incidence in this population to inform power calculations for a future definitive efficacy trial. Additionally, the protocol’s emphasis on harm reduction in both arms ensures that all participants receive overdose prevention services regardless of randomization.

However, several limitations warrant consideration. The single-site design and research setting may limit immediate generalizability, particularly to environments without robust harm reduction infrastructure. Monthly injections could exclude individuals facing significant barriers to regular healthcare engagement, though this mirrors barriers to implementation of other long-acting medications in this population.

This study represents a first step toward expanding the role of medications for primary prevention of opioid overdose. Success of this pilot would demonstrate the acceptability of offering long-acting opioid overdose prevention in settings serving people who use stimulants and provide a scalable model for implementation in similar contexts. Moreover, by targeting a population not eligible for methadone or buprenorphine, this approach could help address a critical gap in current overdose prevention efforts. As the overdose crisis continues to evolve, innovative strategies that account for changing patterns of drug use and market dynamics are increasingly vital.

## Data Availability

No datasets were generated or analysed during the current study.
